# Click chemistry in the development of PROTACs

**DOI:** 10.1039/d3cb00199g

**Published:** 2023-12-29

**Authors:** Ce Yang, Ravi Tripathi, Binghe Wang

**Affiliations:** a Department of Chemistry and Center for Diagnostics and Therapeutics, Georgia State University Atlanta Georgia 30303 USA wang@gsu.edu

## Abstract

Proteolysis-targeting chimeras or PROTACs are hetero-bifunctional molecules designed to mediate the disposal of a target protein *via* recruitment of the ubiquitination–proteasome degradation machinery. Because of the chimeric nature of such molecules, their synthesis requires a key step of “assembling” whether in the lab or *in situ*. Furthermore, targeted PROTACs often are hetero-trifunctional and require a second “assembling” step. Click chemistry has the unique advantages of tethering two or more molecular entities of choice under near physiological conditions and therefore has been applied to the development of PROTACs in various ways. This review provides a succinct summary of this field with a critical analysis of various factors that need to be considered for optimal results. Specifically, we examine issues including applications of click chemistry in *in situ* assembly for improved delivery, conjugation with a targeting group for selectivity, rapid synthesis for linker optimization, and lysosomal degradation of extracellular and membrane-associated proteins. We also examine reaction kinetics issues whenever possible or warranted.

## Abstract 2

This review discusses leveraging click chemistry to address unmet needs in PROTAC development.

## Graphical abstract

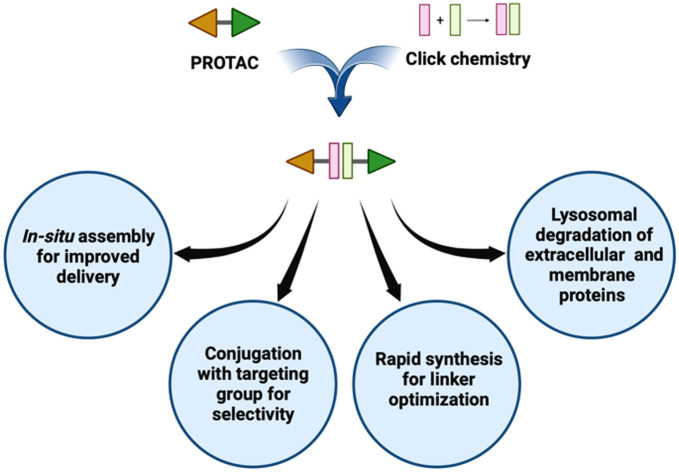

## 1. Introduction

Proteolysis-targeting chimeras (PROTACs) are hetero-bifunctional molecules that constitute a ligand for a target protein tethered to an E3 ubiquitin ligase recruiting group to bring together the ubiquitinating machinery and the target protein for facilitated proteasomal degradation. Different from classical occupancy-driven protein inhibition for therapeutic intervention, PROTACs are event-driven and only require transient target binding to initiate a cascade of events, leading to reduced protein levels. Due to the catalytic nature of their mechanism of action (MOA), PROTACs are potentially more potent than traditional inhibition-based drugs, at least in some applications. Because of this advantage, PROTACs have generated widespread interest since the publication of the first PROTAC molecules by the lab of Prof. Craig Crews.^1^ Furthermore, the first orally-administered PROTAC, ARV-110, is currently in phase II clinical trials, showing the promising potential of this class of therapeutics.^2^ However, PROTACs are hetero-bifunctional molecules and possess molecular weights in a significantly higher range than what is desired for optimal drug-like properties. Therefore, low oral bioavailability and membrane permeability are issues of concern. Furthermore, achieving tissue specificity is a persistent topic for drug development, especially for PROTACs, which lack intrinsic tissue-selectivity and can have potential off-target side effects. Consequently, there is much room for improvement in developing PROTACs as therapeutics, especially as orally active therapeutics.

Click chemistry^3–5^ has found widespread application^6–13^ in a range of areas including in expediting drug discovery and optimization processes. In the context of PROTACs, click reactions have been used for direct conjugation *in vitro* or *in vivo* (see Section 2 for select examples). There have also been reports on strategies of using click reactions to conjugate a targeting moiety for tissue selectivity as shown in Section 3. Moreover, click reactions can also be used for rapid construction of PROTAC libraries (see Section 4 for highlighted examples). Table 1 shows representative examples of click reaction applications in PROTAC development. This review especially focuses on using click chemistry approaches to address PROTACs’ problems in terms of poor permeability, poor tissue-selectivity, and synthetic challenges. We also address targeted degradation of extracellular and membrane-associated proteins in Section 5. Applications were also discussed in the context of reaction kinetics and concentrations used, especially for *in situ* assembly at low to mid-micromolar concentrations, which would require the reaction kinetics to be fast enough for assembly before elimination or metabolism. This is to provide an assessment of the “upper limit” in terms of response time and how reaction kinetics might impact the pharmacological outcome.

**Table 1:** Select examples of click reactions used in PROTAC-related research

Applications	*In situ* assembly for improved permeability	Conjugation with a targeting moiety for tissue selectivity	Rapid construction of PROTAC libraries	Degradation of extracellular proteins
Desirable features of click reactions	1. Compatible with various functional groups.	1. Compatible with various functional groups	1. Compatible with various functional groups	1. Compatible with various functional groups
	2. Fast reaction kinetics in aqueous solution, sufficient for rapid conjugation at μM concentrations	2. Functional in aqueous solution for conjugating with biomacromolecules	2. Fast reaction kinetics	2. Stable for *in vitro* and *in vivo* applications
			3. Compatible with various organic solvents needed for bench reactions	

Examples of applicable click reactions	Strained alkene–tetrazine cycloaddition	Strain-promoted azido–alkyne cycloaddition	Cu-mediated azido–alkyne cycloaddition	Strain-promoted azido–alkyne cycloaddition
	Cu-mediated azido–alkyne cycloaddition	Cu-mediated azido–alkyne cycloaddition	Staudinger ligation	Cu-mediated azido–alkyne cycloaddition
		Tetrazine mediated drug release from a strained alkene		

Kinetic requirements	Reaction rate constant at 100 L mol^−1^ s^−1^ would give the first *t*_1/2_ of 17 min at 10 μM of each reactant.	No specific reaction rate requirement except for the need to complete the reaction within a reasonable period of time at a given concentration.	No specific reaction rate requirement except for the need to complete the reaction within a reasonable period of time at a given concentration.	No specific reaction rate requirement except for the need to complete the reaction within a reasonable period of time at a given concentration.

## 2. Click chemistry approaches for *in situ* assembly of PROTACs for targeting oncogenic proteins

One of the challenges associated with PROTACs is their high molecular weights and the resulting low tissue or cell permeability. To overcome these challenges and improve the tissue/cell permeability of PROTACs, Lebraud *et al.*^14^ examined the idea of *in situ* assembly. In doing so, there is a need for a reaction that is bioorthogonal and fast. The reaction rate needs to be compatible with the low micromolar intracellular concentrations of each PROTAC partner. As analyzed before,^13^ for the assembly to complete within a reasonable period of time (*e.g.*, less than an hour) at low micromolar concentrations, the second-order rate constant needs to be over 100 M^−1^ s^−1^, which gives the first half-life at 17 min at 10 μM and 2.8 hours at 1 μM. In this study, the *trans*-cyclooctene–tetrazine reaction pair was chosen for its fast reaction rate (∼10^4^ L mol^−1^ s^−1^).^10,11^ Briefly, a thalidomide (E3 ubiquitin ligase recruiter) derivative linked to tetrazine and *trans*-cyclooctene-conjugated JQ1 (JQ1–TCO) were synthesized. JQ1 is a ligand for bromodomain-4 (BRD4) with IC_50_ values of 77 nM and 33 nM for BRD4 1 and 2 respectively.^15^ This was intended for delivery of the two partners of the PROTAC molecule separately for improved cellular permeability and intracellular assembly for degradation of BRD4. This approach was termed as CLIPTAC (Fig. 2). To evaluate biological activity, HeLa cells were incubated with varying concentrations of JQ1–TCO for 18 h, followed by treatment with 10 μM Tz–thalidomide for another 18 h. Western blot studies showed that JQ1–TCO induced the degradation of BRD4 in a concentration-dependent fashion. At 3 μM (*t*_1/2_ = 33 s) and 10 μM (*t*_1/2_ = 10 s) of JQ1-TCO together with Tz–thalidomide, complete degradation of BRD4 was noted, while at 0.3 μM (*t*_1/2_ = 5.5 min) and 1 μM (*t*_1/2_ = 100 s) of JQ1-TCO, partial degradation occurred. Then a similar experiment was repeated with varying concentrations of Tz–thalidomide, while the concentration of JQ1–TCO was fixed at 10 μM. A similar trend of BRD4 degradation was observed. BRD4 was completely degraded at high concentrations (3 and 10 μM) and partially at lower concentrations (0.3 and 1 μM). Hence, the CLIPTAC approach demonstrated significant degradation of oncoprotein BRD4 by recruiting cereblon E3 ubiquitin ligase. Therefore, this strategy allows for the use of low molecular weight component molecules to achieve the effects of PROTACs through application of click chemistry. The successful demonstration of the application of click chemistry in PROTAC development spurred additional interest in devising innovative strategies^16–18^ to advance the development of PROTACs.

**Fig. 1:**
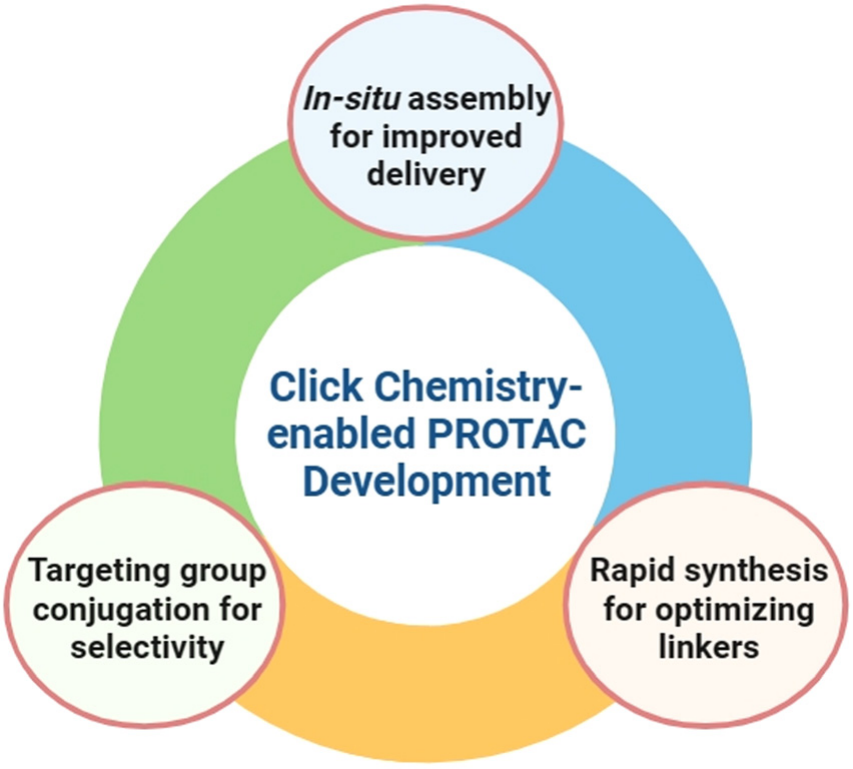
Leveraging click chemistry to address unmet needs in PROTAC development.

**Fig. 2:**
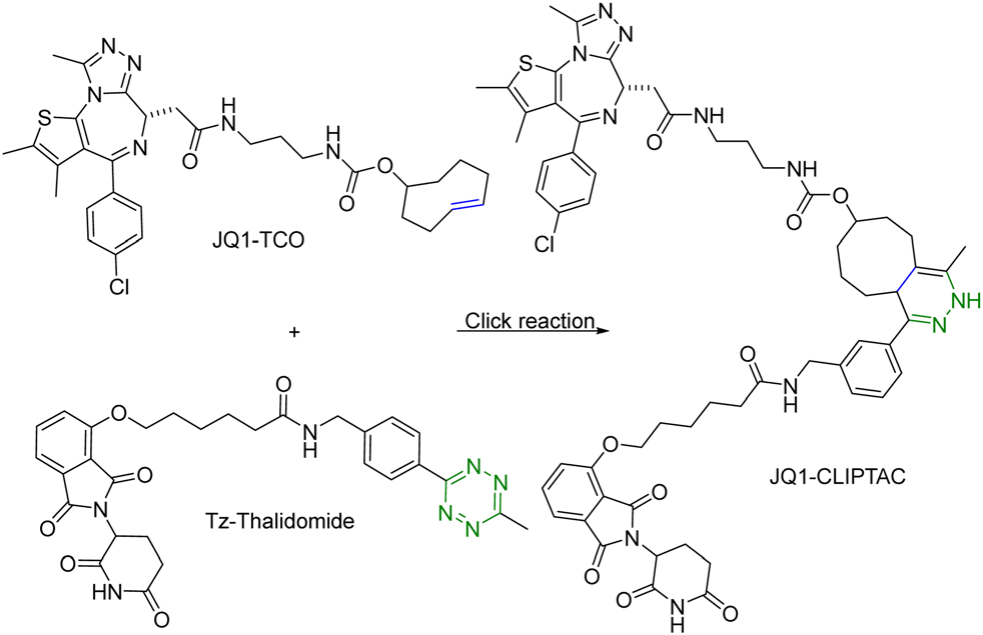
Click reaction between JQ1–TCO and Tz–thalidomide to form JQ1-CLIPTAC for degradation of BRD4 in HeLa cells.

**Fig. 3:**
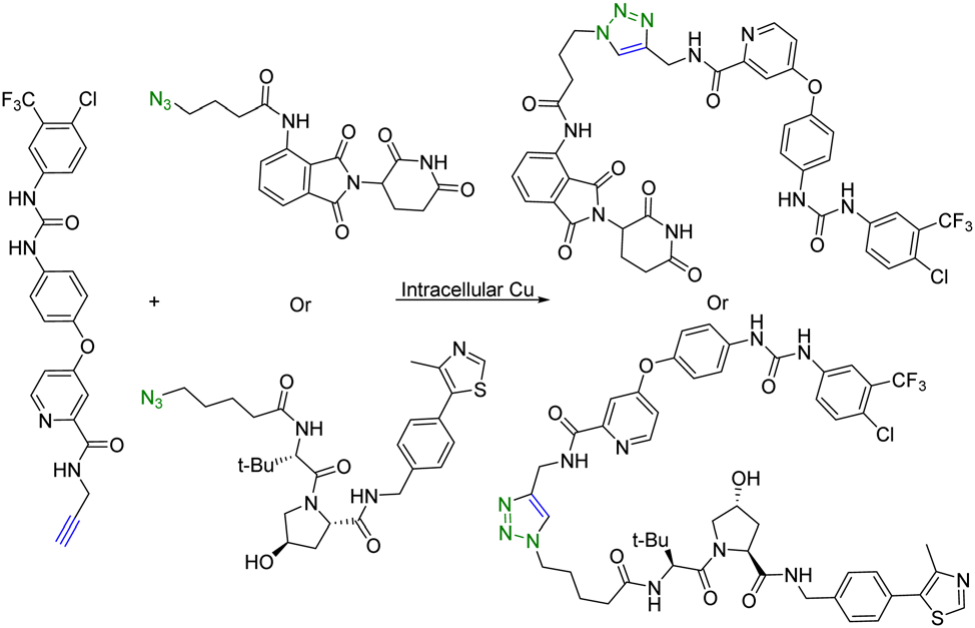
Intracellular self-assembly of PROTACs driven by CuAAC to degrade VEGFR-2 and EphB4 proteins in tumor cells.

Copper (Cu) is an essential micronutrient and plays an important role in cell proliferation and angiogenesis.^19^ Cu concentration was 46% higher in several tumor tissues than in the normal ones.^20^ Owing to significant Cu accumulation in several tumor tissues, tumor-specific therapy based on this property has been developed.^21^ In this regard, Si *et al.*^22^ applied intracellular self-assembled PROTACs with tumor specificity based on Cu-mediated azido-alkyne cycloaddition (CuAAC). Specifically, alkyne-modified sorafenib (targeting the receptor tyrosine kinase ligand) and the azido-modified VHL or cereblon ligand (E3 ubiquitin ligase recruiter) were synthesized for *in situ* assembly of tumor-specific PROTACs using CuAAC (Fig. 3). It is of note that sorafenib is a kinase inhibitor with multiple targets and can inhibit both vascular endothelial growth factor receptor (VEGFR-2) and receptor tyrosine kinase (EphB4).^23^ Furthermore, as determined by HPLC, treatment of tumour cells (A549) or normal cells (HEK293) with a 1:1 mixture of precursor molecules for 24 h generated a chromatographic peak of self-assembled PROTAC. Ultrafast high-resolution mass spectrometry is used to confirm that there are molecular ion peaks of the corresponding self-assembled PROTAC in the cells. More interestingly, self-assembled PROTAC was produced in both A549 and HEK293 cells, but more in A549 cells than in HEK293 cells. Taken together, HPLC and ultrafast high-resolution mass spectrometry results indicated the formation of self-assembled PROTAC intracellularly. Lastly, the cell proliferation inhibitory activity of precursor molecules and the combination of precursor molecules towards A549 and HEK293 cells was measured by MTT assays. The combination of precursor molecules had a significant effect on the cell viability of A549 cells (IC_50_ = ∼1 μM). However, the combination of precursor molecules had no obvious cytotoxicity to HEK293 cells at 1 μM. Altogether, this intracellular self-assembly PROTAC approach opens new avenues for tumor-specific degradation of the target protein based on the fact that concentrations of copper ions are higher in tumor tissues than in normal tissues. It is important to note that the second-order rate constant of the CuAAC is normally in the range of 100 L mol^−1^ s^−1^.^24^ This means that at the concentration used, the *t*_1/2_ is estimated to be 2.8 hours. Understanding these parameters should help maximize the chance for success.

In summary, to overcome the issue of high molecular weight and thus low permeability of PROTACs, *in situ* assembly using click chemistry has been successfully used. Additional considerations may include the assessment of the optimal reaction rate based on the desired concentrations of each component to ensure timely assembly of the eventual PROTAC molecule and/or prevention of premature (extracellular) assembly of the PROTAC partners.

## 3. Click chemistry approaches to conjugate PROTACs with a targeting moiety for tissue selectivity

**Fig. 4:**
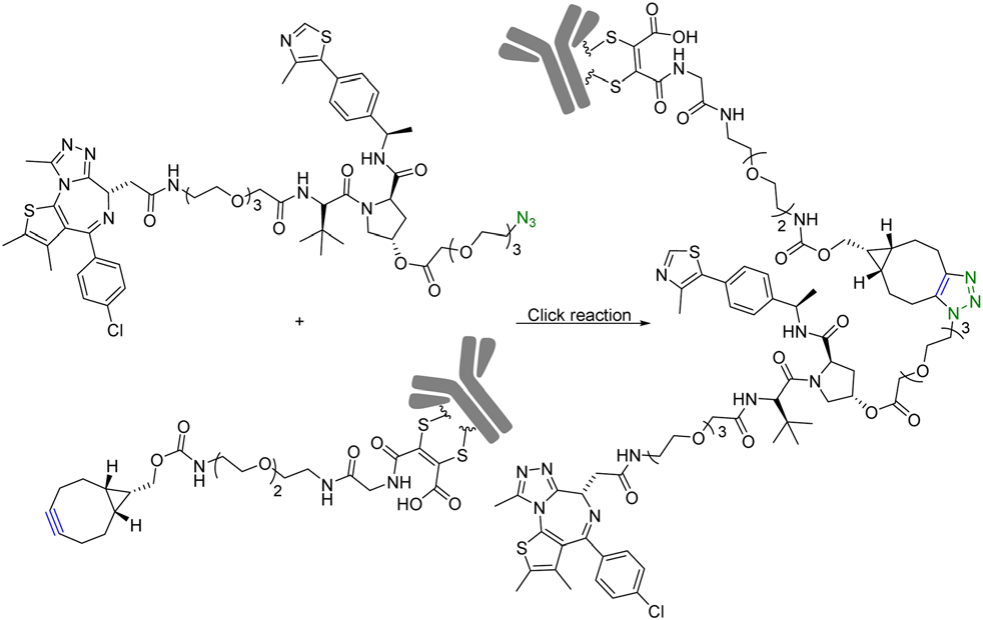
Synthesis of Ab–PROTAC *via* click reaction for degradation of BRD4 in HER2+ cancer cell lines.

Although PROTAC-based approaches do not intrinsically have tissue selectivity, there are ways to achieve added selectivity through conjugation with a targeting molecule. ADCs (Antibody–Drug Conjugates) have gained significant attention in the field of targeted cancer therapy. This targeted approach allows for the selective delivery of the cytotoxic drug to cancer cells while sparing healthy cells and reducing toxicity and side effects. Recognition of cancer cell-specific antigens by ADCs has enabled significant progress in the development of targeted cancer therapy.^25^ The same approach can be applied to the selective delivery of PROTAC molecules. However, antibody conjugation is a complex process even with traditional small molecules. With PROTAC molecules, the conjugation step presents extra challenges because of the size, functional group complexity, and chimeric nature of the molecules. Therefore, such conjugation desires chemistry that fits the profile of click chemistry. Along these lines, Maneiro *et al.*^26^ reported using strain-promoted azido–alkyne cycloaddition (SPAAC) to form antibody-tagged PROTAC molecules for targeted delivery to breast cancer cells, which express human epidermal growth factor receptor 2 (HER2). In this study, an antibody against HER2, trastuzumab, was used as the targeting moiety and the PROTAC molecule used a ligand for the von Hippel–Lindau factor for ubiquitination of the target protein. It should be noted that the von Hippel–Lindau factor recognizes the hydroxyl group of the proline moiety for ubiquitination. As such a ligand for the von Hippel–Lindau factor contains a hydroxyproline moiety, the PROTAC molecule was tethered to an azido-PEG linker by modifying the free hydroxyl group of a von Hippel–Lindau ligand (Fig. 4). Since the presence of a free hydroxyl group is critical for biological activity, its modification is through a cleavable ester bond. It was envisioned that upon trastuzumab-facilitated delivery of the PROTAC to the HER2+ cells, the PROTAC would be released into the lysosomes after ester hydrolysis. Therefore, the free PROTAC subsequently can induce ubiquitination of the target protein. In this case, a ligand JQ1 for BRD4 was used for the purpose of degrading this oncoprotein. With such a design, the azido-modified PROTAC was reacted with BCN-modified trastuzumab through a SPAAC reaction (Fig. 4). It should be noted that in such a chemical synthetic step, biocompatibility is only an issue if antibody stability is affected. In contrast, the reaction rate is less a problem than that in *in situ* PROTAC assembly as described in the previous section. This is because of the high concentration of the reaction components and long reaction time. The biological activity of the antibody–PROTAC (Ab–PROTAC) conjugate was examined against HER2+ cancer cell lines, SK-BR-3 and BT-474. Two HER2− cancer cell lines, MCF-7 and MDA-MB-231, were used as controls. As expected, Western blot studies showed complete degradation of BRD4 by 100 nM Ab–PROTAC after 4 h of incubation in HER2+ cell lines. On the other hand, no degradation of BRD4 was detected in HER2− cell lines. These results show the selective delivery of the PROTAC to the HER2+ cells *via* conjugation with trastuzumab.

**Fig. 5:**
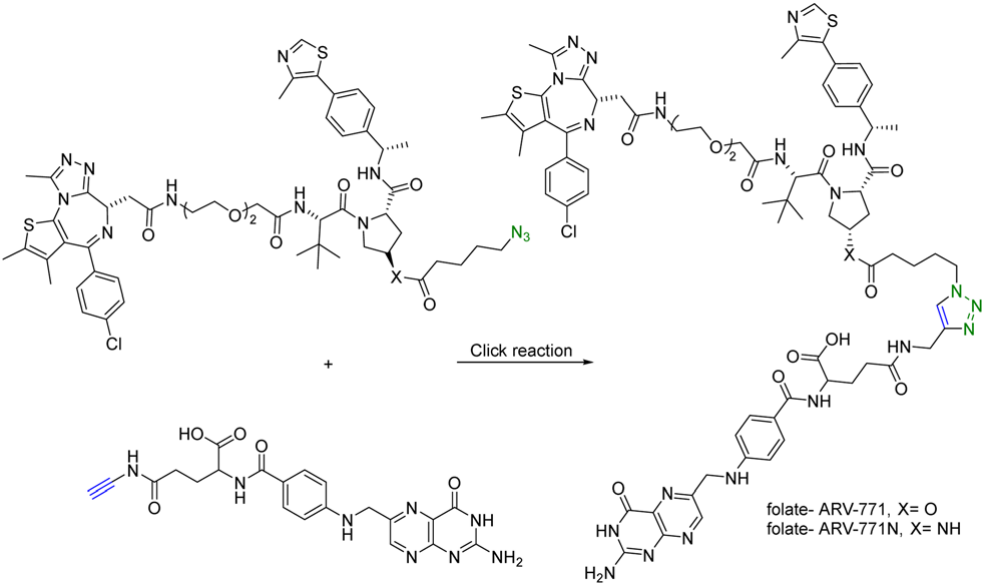
Synthesis of folate conjugated PROTAC *via* click reaction for degradation of BRD4 in FOLR1-expressing cancer cells.

In addition to using an antibody for targeted delivery, one could also use small molecule vectors. For example, folate receptors are overexpressed in various types of cancers, including ovarian, lung, breast, and colorectal cancers. Folate-conjugated drugs exploit this characteristic for targeted drug delivery. The folate moiety acts as a ligand that selectively binds to cancer cells overexpressing the folate receptor, allowing for selective delivery of the drug to the tumour site. However, folate conjugation is chemically challenging for reasons of poor solubility, difficulty in purification, and the need for selective conjugation to the γ-carboxylic acid. Click chemistry offers many of the characteristics suited for such conjugation. Liu *et al.*^27^ synthesized folate-conjugated PROTACs using click chemistry. Since the folate receptor is highly expressed in many types of cancer cells, conjugation with folate enables the selective delivery of PROTACs to cancer cells. ARV-771 is a small-molecule pan-BET degrader based on linking a JQ1 ligand and a von Hippel–Lindau ligand. Specifically, the free hydroxyl group of the von Hippel–Lindau ligand moiety in ARV-771 is linked to 5-azidopentanoic acid through a cleavable ester group. Applying CuAAC, the azido-functionalized PROTAC molecule (ARV-771) was conjugated with folate having an alkyne handle to generate folate–ARV-771 (Fig. 5). In addition, a negative control compound folate–ARV-771N was also prepared by replacing the ester bond with a non-cleavable amide bond. Furthermore, various cancer cell lines (HeLa, OVCAR-8 ovarian cancer cells, and T47D breast cancer cells) exhibiting high levels of folate receptor expression were treated with the folate-conjugated ARV-771 at various concentrations (3, 10, 30, and 100 nM). Significant degradation of the BRD4 oncoprotein in HeLa cells was observed after 12 h incubation with comparable potency to the non-folate-PROTAC counterpart ARV-771. Similar effects were also observed in OVCAR-8, and T47D cell lines. In addition, the negative control, folate-ARV-771N, was incapable of eliciting BRD4 degradation in all the cell lines. Next, these compounds were evaluated against noncancerous cell lines such as HFF-1 human fibroblast cells, HK2 human kidney epithelial cells, and 3T3 mouse fibroblast cells with low levels of folate receptor α (FOLR1) expression. Under the same experimental conditions, ARV-771 induced a significantly higher degradation of BRD4 than both folate–ARV-771 and ARV-771N in noncancerous cell lines. In FOLR1-expressing cancer cell lines, including HeLa cells, OVCAR-8 cells and T47D cells, folate–ARV-771 had a comparable efficiency to ARV-771 in degrading BRDs, while the non-cleavable negative control, folate–ARV-771N did not. In contrast, folate–ARV-771 had a higher cell killing IC_50_ than ARV-771 in noncancerous normal cell lines, such as HFF-1 cells (>10 μM *vs.* 1.1 μM), HK2 cells (2.1 μM *vs.* 166 nM) and 3T3 cells (1.4 μM *vs*. 210 nM). In summary, by utilizing the folate receptor-mediated internalization, folate-conjugated PROTACs can improve the tissue selectivity of PROTACs towards cancer cells, while minimizing potential toxicity/side effects in normal tissues/cells.

**Fig. 6:**
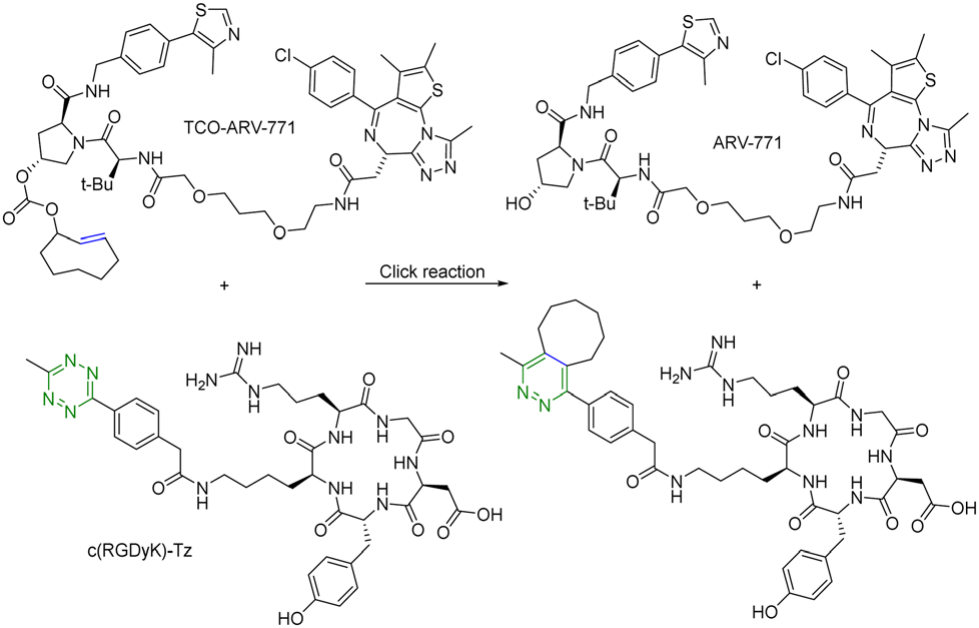
Click and release reaction between TCO–ARV-771 and c(RGDyK)-Tz for degradation of BRD4 in HeLa cells.

For selective delivery, drug targeting can also be achieved through selective activation at the desired site. For this, one can use on-demand activation at the tumor site through click chemistry.^28–30^ It should be noted that for on-site activation, it is critical that the reaction rate for the “activation reaction” is fast so as to give a short *t*_1/2_ at low micromolar concentrations. In one recent example,^31^ a tetrazine-mediated reaction was used for click-and-release prodrug activation. As discussed in the previous section, the *trans*-cyclooctene-tetrazine reaction is very fast with a second-order rate constant on the scale of ∼10^4^ L mol^−1^ s^−1^.^10,11^ Briefly, an inactive PROTAC prodrug TCO–ARV-771 was synthesized by conjugating a ligand of von Hippel–Lindau E3 ligase ubiquitin ligase with a *trans*-cyclooctene (TCO) group. A tetrazine (Tz)-modified RGD peptide, c(RGDyK)-Tz, was designed for targeting the integrin α_v_β_3_ biomarker in cancer cells and for selectively activating TCO–ARV-771 to release ARV-771 (Fig. 6). The biological activity of TCO–ARV-771 and c(RGDyK)-Tz was assessed against HeLa cells that overexpress the α_v_β_3_ integrin. Complete degradation of BRD4 was observed after 3 h treatment with 400 nM TCO–ARV-771 and 1.0 μM c(RGDyK)-Tz. However, TCO–ARV-771 itself did not induce BRD4 degradation in HeLa cells at various concentrations (100–400 nM). Similarly, antiproliferative effects of TCO–ARV-771 and c(RGDyK)-Tz against HeLa cells respectively indicated much lower cell killing propensities (IC_50_ of TCO–ARV-771 is 4.45 μM and IC_50_ of c(RGDyK)-Tz is more than 10 μM). However, co-treatment of TCO–ARV-771 and c(RGDyK)-Tz has cytotoxicity (IC_50_ = 389 nM) similar to ARV-771 (IC_50_ = 466 nM). These results indicate that the inactive PROTAC prodrug TCO–ARV-771 is selectively activated in cancer cells due to the presence of the α_v_β_3_ integrin binding ligand c(RGDyK) in the activating agent c(RGDyK)-Tz. The click-and-release strategy has provided medicinal chemists with a powerful tool to achieve the targeted delivery of PROTACs through easy and efficient conjugation of small vector molecules or monoclonal antibodies. It is worth mentioning that TCO–tetrazine ligation can also be used for on-demand termination of PROTACs.^32^

In summary, it is important to note that off-tumour toxicity is a significant consideration in the development of PROTACs. Researchers continuously work towards utilizing click chemistry to conjugate PROTACs with a targeting molecule for selective delivery to cancer cells. A click-and-release approach provides a targeted approach. Ongoing research^33^ continues to refine the design and delivery of PROTACs using click chemistry to enhance their efficiency and broaden their applications in cancer therapy.

## 4. Click chemistry approaches for rapid synthesis of PROTACs and efficient construction of PROTAC libraries

A PROTAC requires two binding moieties, one for an E3 ubiquitin ligase and the other for the protein of interest. Most designs rely on the use of known ligands with the key chemistry development being the optimization of the conjugation step. This seemingly straightforward issue (linker design) requires quite a bit of careful work. Specifically, the design of the linker is a critical aspect that can influence the potency of the PROTAC molecule. The length and flexibility of the linker play a crucial role in the potency of PROTACs. Linkers that are too short or rigid may not provide enough spatial freedom for the PROTAC and E3 ligase to interact optimally with their respective targets, potentially leading to reduced binding affinity and degradation efficiency. Conversely, linkers that are too long or flexible can increase the chances of off-target interactions. The chemical composition of the linker can also affect the stability, solubility, and pharmacokinetic properties of PROTACs. Linkers should be designed to maintain stability under physiological conditions while ensuring sufficient solubility for effective administration. Different target proteins may have varying requirements for linker design due to differences in their structures and binding sites. Furthermore, an optimal linker for one PROTAC may not work for another. Therefore, there is linker optimization work for each, which requires quite a bit of synthetic efforts. As a result, an efficient “linking” approach would help facilitate the optimization work. Click chemistry seems to be ideally suited for such applications.

**Fig. 7:**
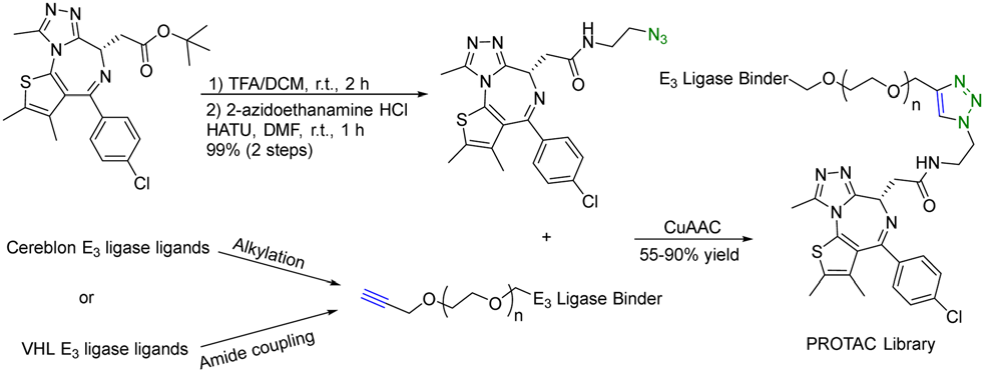
A rapid CuAAC synthesis platform for PROTAC for degradation of BRD4 in the NCI-H661 cell line.

There are many successful examples of using click chemistry in developing PROTACs. For example, in 2017, a rapid synthesis platform of bispecific molecules for PROTAC based on CuAAC was developed.^34^ As shown in Fig. 7, CuAAC couples the BRD4 ligand JQ1 with an azido moiety and the cereblon E3 ligase ligand with an alkyne moiety forming stable triazole rings to generate a PROTAC library. Briefly, a ligand (JQ-1) for the bromodomain and extra terminal domain-4 (BRD4) was used as the target protein ligand and converted into an amide containing azido moiety for CuAAC as shown in Fig. 7. For the E3 ligase ligand portion, cereblon and VHL ligase ligands were used and converted into terminal alkyne motifs containing varying ethylene glycol units. Ten new PROTACs were synthesized in 55–90% yield. To evaluate the activity of these PROTACs, a cell-free amplified luminescence proximity homogeneous assay was developed to determine if a ternary complex was formed among the BRD4 protein, PROTAC, and the E3 ligase. In this assay, the DC_50_ value of the most active IMiD (immunomodulatory imide drug)-derived PROTAC is 0.20 μM, similar to the benchmark IMiD PROTAC's DC_50_ value of 0.14 μM. In the BRD4/PROTAC/VHL proximity assay, the VHL PROTACs were formed by click ligation. These VHL/BRD4 PROTACs showed low double-digit nanomolar activities. However, the presence of the triazole motif in the linker caused a small decline in solubility and cLogP. Furthermore, a series of control experiments were performed with the NCI-H661 cell line. The VHL/BRD4 PROTACs resulting from the click ligation were used for dosing of this cell line. Dosing these compounds at 1 μM after 4 h resulted in more than 90% degradation of BRD4 *vs.* a DMSO control. Co-treatment with cereblon or VHL ligands resulted in the blockage of PROTAC-induced degradation of BRD4. No appreciable degradation resulted from individual treatment or co-treatment of the azide/alkyne components, confirming that nonlinked CuAAC components of the PROTACs were not sufficient for degradation. In short, CuAAC can be used to prepare the PROTAC library rapidly, and the degradation ability of this PROTAC library can be measured by a proximity assay efficiently.

**Fig. 8:**
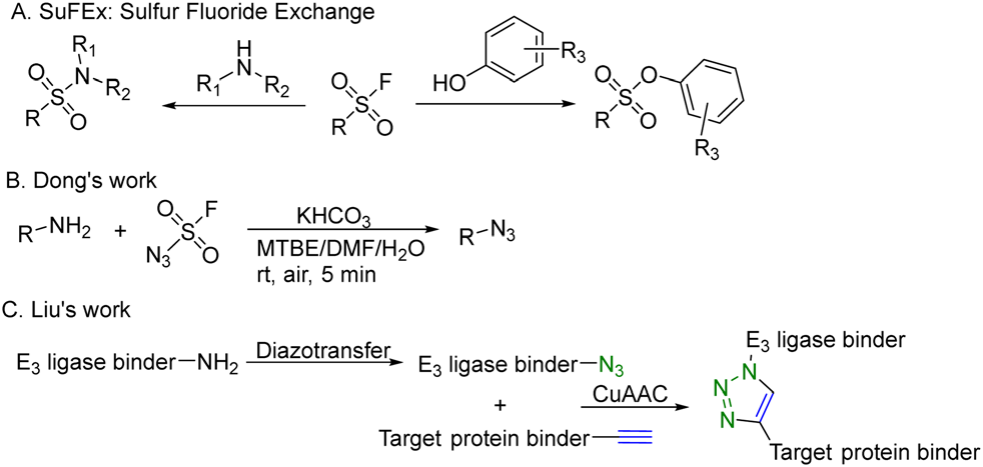
Construction of a PROTAC library using a diazotransfer and CuAAC domino method for degradation of BCR-ABL fusion protein or BRD4 in leukemia cells.

CuAAC is widely used in rapid conjugation applications.^4^ However, appropriate azide-containing compounds are not always available for a particular application. Meanwhile, the potential of other click reactions has also been explored. Sharpless and colleagues introduced the sulfonyl fluoride exchange (SuFEx) click reaction in 2014 (Fig. 8A).^35^ Unlike other click reactions, SuFEx is not a cycloaddition process and involves a diverse range of chemical transformations. While exploring new SuFEx reaction modules, Dong's lab found an unexpected route that fluorosulfuryl azide diazotizes primary amines to form azides at room temperature (Fig. 8B).^36^ This diazotransfer reaction is fast, high-yielding and selective, fulfilling the criteria to be categorized as the click reaction.^3^ In 2021, Liu and colleagues took advantage of this diazotransfer reaction and CuAAC to construct a cereblon E3 ligase ligand-based azide library for PROTAC research.^37^ As shown in Fig. 8C, the existing cereblon (CRBN) recruiter amine library was converted to a wide array of azides by fluorosulfuryl azide, fastly and modularly. The reaction of compounds in the azide library with the corresponding alkynyl-tagged POI ligands *via* CuAAC led to the construction of a series of analogues. To test this approach, two kinds of cereblon E3 ligase ligands were used to perform diazotransfer under mild conditions by using fluorosulfuryl azide. Fluorosulfuryl azide is much safer than sodium azide and fluorosulfuryl azide is a commercially available diazotizing reagent and the azide source. A range of pomalidomide- and lenalidomide-derived azides with different lengths of PEG or full carbon chains were acquired in moderate to high yields. Then, these CRBN ligand-derived azides were used to verify the idea of construction of a PROTAC library. Pomalidomide-based azides were linked with alkynyl-tagged dasatinib derivatives. Dasatinib is a tyrosine kinase inhibitor binding with a chronic myelogenous leukemia-related BCR-ABL fusion protein. These new PROTACs displayed an even stronger activity than the reported BCR-ABL PROTAC with concentrations as low as 10 nM. Such a one-pot approach was also applied by linking two pomalidomide-based amines with JQ1. Two new BET degraders were made by this one-pot method and displayed 8- or 11-fold higher antiproliferative effects than dBET1, one of the known BET degraders with high efficacy.^38^ Taken together, these results indicate that this diazotransfer and CuAAC domino method can overcome the limitations of azide synthesis and can be used to rapidly prepare a library in search of efficient degraders.

**Fig. 9:**
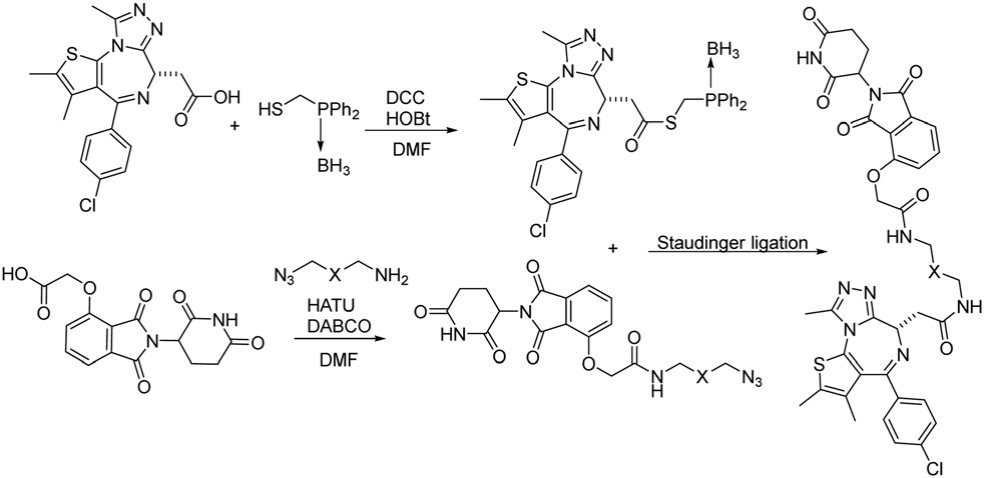
Construction of PROTACs using traceless Staudinger ligation for degradation of BRD4.

Besides CuAAC and SPAAC, Staudinger ligation has also been widely used for bioconjugation.^39–41^ In 2020, Burkart and coworkers reported a parallel, one-pot assembly approach for PROTACs by using traceless Staudinger ligation.^42^ As shown in Fig. 9, the general process began with the *in situ* formation of a thioester from JQ1 acid. The thioester can be purified and stored under dry conditions. Then starting with thalidomide acid and HATU, the linker amine and DABCO were added as solutions in DMF. The resulting azides were then coupled with the JQ1 thioester to yield a second amide bond through traceless Staudinger ligation. The Staudinger ligation was initiated yielding the PROTAC in a one-pot fashion. LC-MS analysis indicated that the PROTAC was obtained in 48% yield. Overall, traceless Staudinger ligation utilizes activated esters generated *in situ* and enables parallel synthesis of PROTAC linker variants.

One of the challenges in PROTAC discovery is the need to increase throughput and improve the quality of PROTAC libraries. Click chemistry is a versatile toolbox for this purpose and provides substantial benefits in accelerating PROTAC discovery and optimization. Click chemistry allows the speedy construction of PROTAC libraries and screening of potent degraders more quickly.

## 5. Beyond PROTACs: click chemistry approaches to conjugate an antibody with a ligand for a cell-surface lysosome-shuttling receptor for degradation of extracellular and membrane-associated proteins

**Fig. 10:**
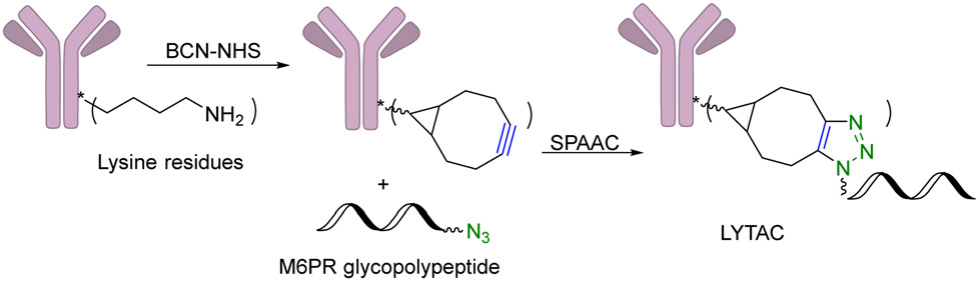
Construction of a LYTAC using SPAAC for degradation of EGFR in HeLa cells.

Another limitation of PROTACs is the requirement for the protein target to be intracellular as the proteasome is the degrading machinery. However, extracellular and transmembrane proteins account for 40% of all proteins, and many of them are related to cancer or other diseases. To expand the application of PROTAC-type molecules, lysosome-targeting chimeras (LYTACs) have been reported for the degradation of extracellular proteins.^43^ For proof-of-concept studies, a protein-targeting moiety was linked with a glycopeptide ligand that binds and activates a cell-surface lysosome-shuttling receptor *via* SPAAC. Specifically, an anti-mouse IgG was used for targeting. As shown in Fig. 10, lysine residues in a polyclonal anti-mouse lgG are non-specifically labeled with bicyclononyne-*N*-hydroxysuccinimide (BCN-NHS) and then conjugated *via* SPAAC with an azido-terminated glycopolypeptide, which binds to a cation-independent mannose-6-phosphate receptor (CI-M6PR). Incubation of K562 cells with this LYTAC and mouse IgG labeled with Alexa Fluor-488 (AF488) led to a 40-fold increase in the lysosomal AF488 signal compared to non-M6Pn-bearing antibodies, indicating efficient shuttling of IgG molecules to the lysosome. Furthermore, this strategy was expanded to apolipoprotein E4 (ApoE4), which is implicated in neurodegenerative diseases. SPAAC is used for linking an antibody that recognizes ApoE4 with a glycopolypeptide that recognizes CI-M6PR. After incubating this LYTAC with K562 cells for 8 h, a 13-fold increase in the uptake of ApoE4 was observed. An EGFR-targeted LYTAC was also made by SPAAC by linking the glycopolypeptide with cetuximab (an EGFR-blocking antibody). After 24 h incubation of this LYTAC with HeLa cells, greater than 70% degradation of EGFR was observed. In short, these bifunctional lysosome-targeting chimeras can degrade extracellular proteins and overcome the limitation of PROTACs in requiring the target protein to be intracellular.

**Fig. 11:**
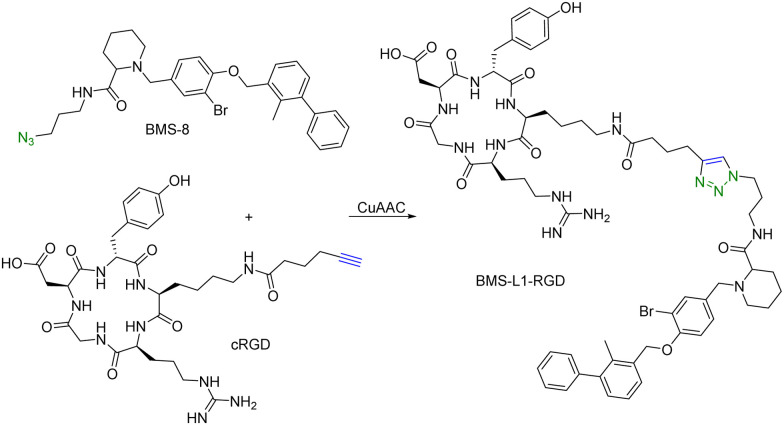
Construction of an integrin-facilitated molecular degrader using CuAAC for degradation of PD-L1 in tumor cells.

Many tumour-related proteins are membrane-associated. For example, programmed death-ligand 1 (PD-L1) is a membrane-associated protein and is overexpressed in many tumour cells. To degrade PD-L1, there is a need for developing new approaches. In 2022, Fang and colleagues developed integrin-facilitated molecular degraders to degrade membrane-associated proteins.^44^ As shown in Fig. 11, an azido derivative of BMS-8, a small molecule inhibitor targeting PD-L1 with high affinity, was linked with the integrin-recognition motif cRGD by CuAAC. This bifunctional compound BMS-L1–RGD can bind both PD-L1 and integrin on the cell surface. After recruiting PD-L1 and integrin by BMS-L1–RGD, PD-L1 is shown to enter cells with integrin through endocytosis. Protein degradation is then triggered by the lysosomes. Since PD-L1 is an immunomodulator on the cell membrane, degradation of PD-L1 leads to enhanced immunotherapy for cancer. To test this approach, MDA-MB-231 breast cancer cells with a high expression level of PD-L1 were treated with BMS-L1–RGD. 70% of PD-L1 were degraded within 8 h in cells treated with 25 nM BMS-L1–RGD. To confirm that the degradation mechanism was *via* lysosomes, MDA-MB-231 cells were incubated with BMS-L1–RGD in the presence or absence of a lysosome inhibitor bafilomycin or a proteasome inhibitor MG132. The degradation of PD-L1 was inhibited by bafilomycin but not by MG132, indicating that the degradation is lysosome-mediated. To verify the degradation mechanism mediated by integrin, MDA-MB-231 cells were preincubated with an excess amount of cRGD to block the integrins on the cell surface and then treated with BMS-L1–RGD. After pretreatment with excess cRGD, BMS-L1–RGD showed no PD-L1 degradation, confirming the integrin-dependent nature of PD-L1 degradation. Furthermore, the antitumor effects of BMS-L1–RGD were evaluated in a C57BL/6J mouse model with B16F10 tumour xenograft (5 mg kg^−1^ BMS-L1–RGD, every other day, i.v.). The control group was treated with an equimolar amount of BMS-8. After being dissected and weighed, the tumour volume (500 mm^3^) of the BMS-L1–RGD-treated mice were found to be much smaller compared to the control group (2000 mm^3^). Tumour weight in mice treated with BMS-L1–RGD was less than 1 g and significantly smaller than that of the control group (2 g). In summary, bifunctional compounds resulting from the CuAAC of the PD-L1 ligand with an integrin-recognition ligand can degrade PD-L1 both *in vitro and in vivo*, expanding the target protein degradation toolbox for membrane-associated proteins.

In summary, given their unique MOA through the intracellular ubiquitin–proteasome system, PROTACs are only able to target intracellular proteins. However, the potential use and feasibility of click chemistry for lysosomal degradation of extracellular and membrane-associated proteins provide a powerful solution to overcome the drawbacks of PROTACs.

## 6. Future developments and challenges

**Fig. 12:**
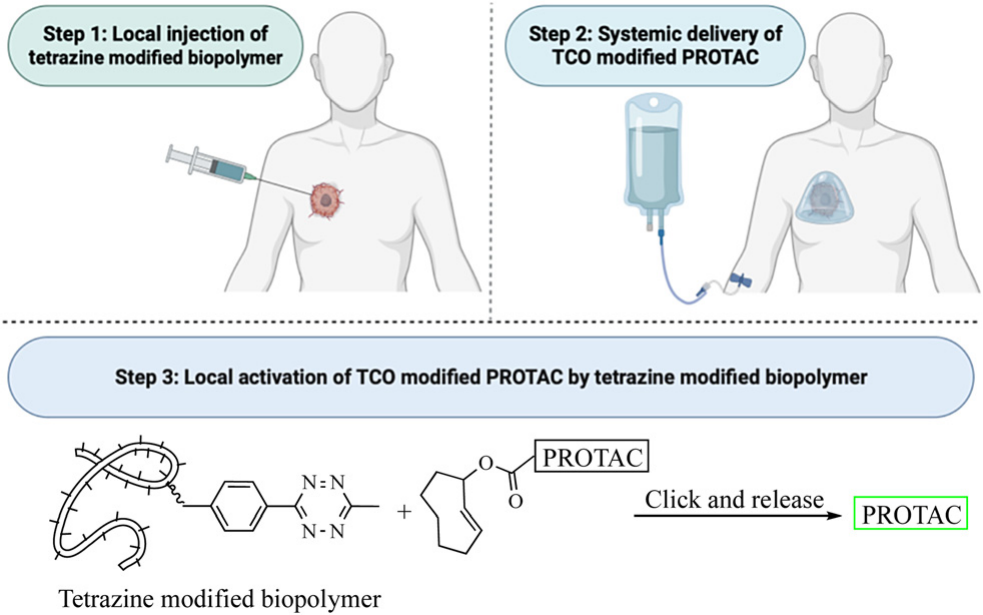
Proposed click-and-release PROTAC through local capture and activation.

This review critically summarizes the literature on different types of successful applications of click chemistry in developing PROTACs and their *in vitro* studies. However, for future development, it is very important to recognize that there are extra layers of challenges for success in *in vivo* studies. Issues including metabolic stability,^45^ redistribution, and clearance^46^ of the click reagents all need to be considered. For example, if the reaction rate is too fast, the click reaction can happen before the reagents reach the target tissue. This likely will lead to reduced effective concentrations and cause off-target effects *in vivo*. If the reaction rate is too slow, click reagents will likely be cleared before the desired click reaction in the target tissue. Then, the click product may never reach the threshold concentration needed for efficacy. Achieving the right balance will require extensive work in animal models. Furthermore, extrapolating the results from animal models to humans may also represent a significant challenge because allometric scaling is not a trivial issue even for regular small molecule drugs. For PROTACs that require *in situ* assembly, the degree of difficulty is much higher. In 2021, Royzen and co-workers^47^ described a click-and-release drug delivery platform through local capture and activation. In step 1, a tetrazine-modified biopolymer is locally injected into the target tissue. The covalent linkage with the biopolymer causes no/minimal diffusion of the tetrazine agent. In step 2, a TCO-modified drug is infused systemically. The locally deposited tetrazine agent enables localized click activation of the prodrug in the target tissue. This platform represents one way for further development of PROTACs based on click activation *in vivo* (Fig. 12).

## 7. Conclusion

Since their first report in 2001, applications of PROTAC approaches have experienced rapid growth. However, there are still challenges ahead. Click chemistry is ideally suited to help overcome the remaining challenges. In this review, we list various examples of leveraging click chemistry to address these challenges. We also discuss using click chemistry to expand targeted protein degradation beyond PROTACs. Collectively, this review summarized recent applications of click chemistry in the development of PROTACs. We hope that this review will stimulate more research on applying click chemistry in PROTAC development.

## Author contributions

C. Y. and R. T. each drafted two sections of the manuscript. C. Y. coordinated the preparation of individual sections and participated in revising the manuscript. B. W. conceived the idea for the manuscript, provided overall guidance during the preparation process, and revised the entire manuscript.

## Conflicts of interest

There are no conflicts to declare.

## Supplementary Material
